# Supplementary material for: Prognosis Factors of Patients Undergoing Renal Replacement Therapy
Source: J Pers Med. 2023 Mar 30;13(4):605. doi: 10.3390/jpm13040605 (PMC10141530; doi:10.3390/jpm13040605)

SUPLEMENTAL MATERIAL

Table S1: Bivariate analysis of factors related to renal transplantation.

	Renal transplantation N= 3,776		No renal transplantation N= 7,735		p value
	**N (%)**	**CI 95%**	**N (%)**	**CI 95%**	
**Sex**					
Male	2405 (63.7)	(62.1; 65.2)	4853 (62.4)	(61.3; 63.5)	0.184
Female	1371 (36.3)	(34.8; 37.9)	2922 (37.6)	(36.5; 37.7)	
**Occupational Status**					
Active	925 (48.0)	(45.8; 50.2)	308 (7.7)	(6.9; 8.6)	<0.001
Inactive	1003 (52.0)	(49.8; 54.2)	3674 (92.3)	(91.4; 93.1)	
**Comorbidities**					
Median Charlson index score	3.8	(3.8; 3.9)	7.0	(7.0; 7.1)	<0.001
Diabetes mellitus	979 (21.1)	(19.8; 22.4)	3646 (46.9)	(45.8; 48.0)	<0.001
Congestive heart failure	175 (4.6)	(4.0; 5.3)	2009 (25.8)	(24.9; 26.8)	<0.001
Myocardial infarction	217 (5.7)	(5.0; 6.5)	1849 (23.8)	(22.8; 24.7)	<0.001
Peripheral vascular disease	175 (4.6)	(4.0; 5.3)	1562 (20.1)	(19.2; 21.0)	<0.001
COPD	188 (5.0)	(4.3; 5.7)	1218 (15.7)	(14.9; 16.5)	<0.001
Solid tumor localized	124 (3.3)	(2.8; 3.9)	907 (11.7)	(11.0; 12.4)	<0.001
Cerebrovascular accident	139 (3.7)	(3.1; 4.3)	844 (10.9)	(10.2; 11.6)	<0.001
Connective tissue disease	169 (4.5)	(3.9; 5.2)	307 (3.9)	(3.5; 4.4)	0.181
Peptic ulcer disease	75 (2.0)	(1.6; 2.5)	297 (3.8)	(3.4; 4.3)	<0.001
Mild Liver disease	76 (2.0)	(1.6; 2.5)	312 (4.0)	(3.6; 4.5)	<0.001
Moderate to severe liver disease	45 (1.2)	(0.9; 1.6)	150 (1.9)	(1.6; 2.3)	0.004
Leukemia/lymphoma	9 (0.2)	(0.1; 0.4)	166 (2.1)	(1.8; 2.5)	<0.001
Dementia	2 (0.1)	(0.0; 0.2)	196 (1.9)	(1.6; 2.2)	<0.001
Solid tumor metastatic	2 (0.1)	(0.0; 0.2)	115 (1.5)	(1.2; 1.8)	<0.001
HIV	18 (0.5)	(0.3; 0.7)	78 (1.0)	(0.8; 1.2)	0.003
AIDS	4 (0.1)	(0.0; 0.3)	19 (0.2)	(0.2; 0.4)	0.117
HBV	38 (1.0)	(0.7; 1.4)	101 (1.3)	(1.1; 1.6)	0.176
HCV	75 (2.0)	(1.6; 2.5)	296 (3.8)	(3.4; 4.2)	<0.001
**End stage kidney disease etiology**					<0.001
Diabetic Nephropathies	587 (15.5)	(14.4; 16.7)	2245 (28.9)	(27.9; 29.9)	
Glomerular disease	944 (25.0)	(23.6; 26.4)	803 (10.3)	(9.7; 11.0)	
Hypertension and renovascular disease	340 (9.0)	(8.1; 9.9)	1344 (17.3)	(16.5; 18.1)	
Renal tubulo-interstitial nephropathy	400 (10.6)	(9.6; 11.6)	822 (10.6)	(9.9; 11.3)	

	Renal transplantation N= 3,776		No renal transplantation N= 7,735		p value
	N (%)	CI 95%	N (%)	CI 95%	
Familial / hereditary nephropathies	669 (17.7)	(16.5; 19.0)	302 (3.9)	(3.5; 4.3)	
Systemic diseases affecting the kidney	47 (1.2)	(0.9; 1.6)	342 (4.4)	(4.0; 4.9)	
Miscellaneous renal disorders	70 (1.9)	(1.5; 2.3)	235 (3.0)	(2.7; 3.4)	
Chronic renal failure; etiology uncertain	719 (19.0)	(17.8; 20.3)	1682 (21.6)	(20.9; 23.2)	
**End stage kidney disease follow-up**					
Without previous follow up	334 (8.8)	(8.0; 9.8)	1207 (15.5)	(14.7; 16.3)	0.002
ESKD ≤ 6 month of follow-up	531 (14.1)	(13.0; 15.2)	1012(13.0)	(12.3; 13.8)	
ESKD > 6 month of follow-up	2911 (77.1)	(75.7; 78.4)	5556 (71.5)	(70.4; 72.5)	
**Renal replacement therapy at start**					
Median age onset RRT	51.0	(50.6; 51.5)	68.2	(68.0; 68.6)	<0.001
Programed	3014 (80.7)	(79.4; 82.0)	4740 (61.5)	(60.4; 62.6)	<0.001
Not programed	720 (19.3)	(18.0; 20.6)	2963 (38.5)	(37.4; 39.6)	
**Vital Status**					
Age at death (median)	65.4	(64.4; 66.5)	73.3	(73.0; 73.6)	<0.001
Alive	3413 (90.4)	(89.4;91.3)	3238 (41.6)	(40.6;42.7)	<0.001

Figure S1: ROC curve of multivariate analysis.

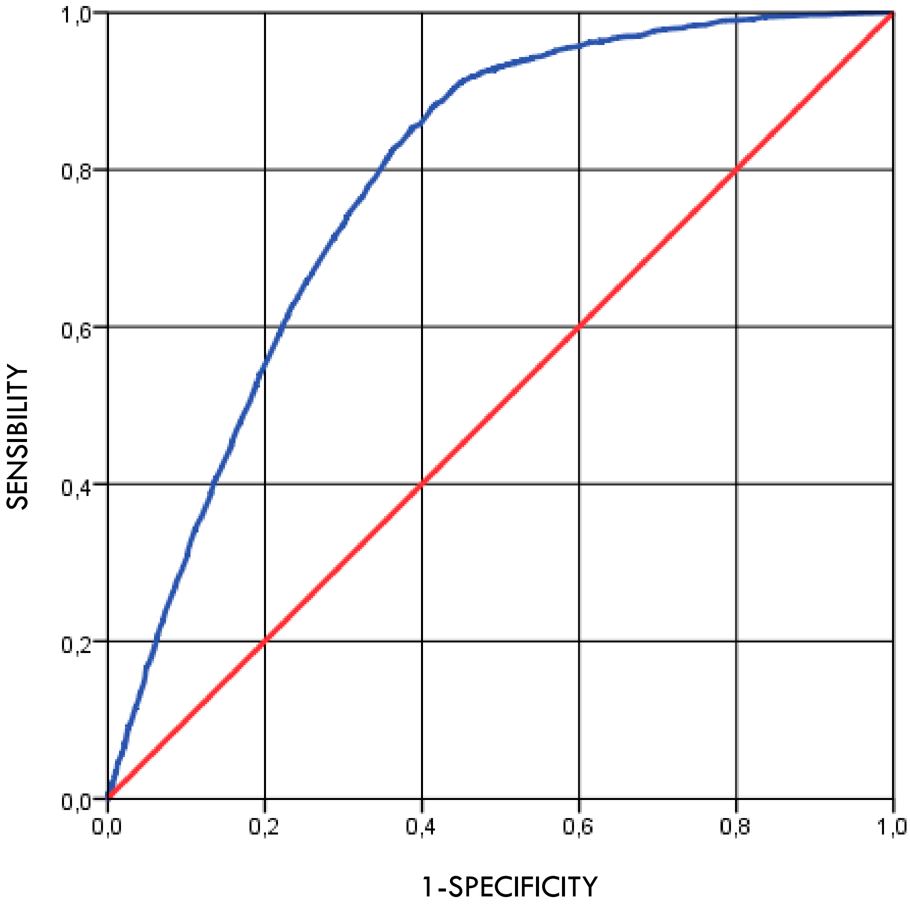

Supplement: Supplementary file 1 [file jpm-13-00605-s001.zip › jpm-2316327-supplementary.pdf]
